# Transient Arginine Vasopressin Deficiency Induced by Valproic Acid Intoxication: A Case Report

**DOI:** 10.7759/cureus.65165

**Published:** 2024-07-23

**Authors:** Taichi Kato, Shunki Hiyama, Mirai Sano, Gen Nakamura, Kazuhiro Sugiyama

**Affiliations:** 1 Tertiary Emergency Medical Center, Tokyo Metropolitan Bokutoh Hospital, Tokyo, JPN

**Keywords:** central diabetes insipidus (cdi), desmopressin, l-carnitine, arginine vasopressin deficiency, valproic acid overdose

## Abstract

Valproic acid is commonly used for treating seizures and psychiatric disorders. Valproic acid is a common anticonvulsant drug causing overdose for suicidal purposes. The most common symptom of valproic acid poisoning is central nervous system damage. Most cases result in mild to moderate drowsiness, while in severe cases, fatal cerebral edema and coma have been reported. Other complications include respiratory depression, hepatotoxicity, thrombocytopenia, and multi-organ failure resulting in circulatory collapse. Herein, we present a case of a 42-year-old woman who ingested an overdose of 600 mg nitrazepam, 50 mg olanzapine, and 35,600 mg valproic acid. The maximum daily doses for nitrazepam, olanzapine, and valproic acid are 15, 20, and 1200 mg, respectively. This overdose led to reversible arginine vasopressin (AVP) deficiency as a rare but significant complication. The deficiency led to polyuria with dilute urine, which was effectively suppressed by AVP administration. This case highlights the potential for reversible AVP deficiency as a rare but significant complication of valproic acid overdose. Early diagnosis and appropriate management are crucial for favorable outcomes.

## Introduction

Valproic acid is used to manage seizures and various psychiatric disorders. It is a simple branched-chain carboxylic acid absorbed from the gastrointestinal tract, reaching peak blood concentrations within one to four hours [1]. Additionally, valproic acid is the common anticonvulsant drug causing overdose for suicidal purposes, with 41.6 reports of overdose per 100,000 people reported by the AAPCC in 2006-2019 [2]. Valproic acid sodium metabolism increases propionic acid and valproyl-CoA. The activity of carbamoyl phosphate synthetase I (CPS-I), a crucial enzyme in the urea cycle, is inhibited as a result. Consequently, carnitine decreases, impairing the uptake of medium-chain fatty acids into mitochondria and inhibiting β-oxidation, leading to increased ammonia levels [1]. In cases of hyperammonemia, supplementation with L-carnitine may be considered [3]. Despite its high protein binding rate of 90%, this binding decreases at higher concentrations, allowing the drug to be removed via blood purification techniques [4]. Treatment strategies such as the administration of meropenem have been reported to lower valproic acid blood concentrations [5,6]. Blood concentrations exceeding 850 μg/mL of valproic acid have been reported to induce coma in all cases [1]. The literature documents only a single case of arginine vasopressin (AVP) deficiency caused by valproic acid, highlighting the rarity of this complication. AVP, also known as antidiuretic hormone (ADH), is crucial for regulating water balance in the body by promoting water reabsorption in the kidneys. The deficiency of AVP leads to impaired water reabsorption in the kidneys, resulting in the excretion of large volumes of dilute urine, known as polyuria [7]. In this report, we present a unique case of AVP deficiency that resolved with a reduction in valproate blood concentration, leading to the alleviation of diabetes insipidus. The AVP deficiency caused by this overdose manifested as polyuria with dilute urine, effectively managed through AVP administration.

## Case presentation

A 42-year-old woman with a history of depression had been prescribed chlorpromazine hydrochloride 50 mg, two tablets; nitrazepam 5 mg, two tablets; flunitrazepam 1 mg, two tablets; and valproic acid 200 mg, one tablet. The patient was last confirmed to be in a normal state at approximately 8:00 PM on the day before admission. At approximately 9:00 PM on the same day, she ingested an overdose of medications, including 600 mg of nitrazepam (120 tablets of 5 mg each), 50 mg of olanzapine (20 tablets of 2.5 mg each), and 35,600 mg of valproic acid (178 tablets of 200 mg each). At 10:00 PM, her family found her to be drowsy and decided to monitor her condition. The next day, at approximately 10:00 AM, the family found her unresponsive to verbal stimuli and called for emergency assistance.

Upon arrival at the hospital, the vital signs of the patient were as follows: GCS E1V1M1, pupils 1.5/1.5 mm and non-reactive, respiratory rate of 20 breaths per minute, heart rate of 76 beats per minute, blood pressure of 83/53 mmHg, and SpO2 of 87% with 10 L of oxygen via a reservoir mask. Arterial blood gas results indicated a pH of 7.272, pO2 of 89.2 mmHg, pCO2 of 49.2 mmHg, HCO3 of 23 mEq/L, and lactate of 2.6 mmol/L. Hypoxaemia and respiratory acidosis were present. Hypotension was also present, and mildly elevated lactate levels were observed. Additional blood test results upon admission are summarized in Table 1.

**Table 1:** Laboratory test results of the patient AST, aspartate aminotransferase; ALT, alanine aminotransferase

Laboratory test	Result	Reference value
White blood cells (×10^3^/μL)	11.3	3.3-8.6
Red blood cells (×10^6^/μL)	3.86	3.86-4.92
Hemoglobin (g/dL)	12.2	11.6-14.8
Platelets (×10^3^/μL)	267	158-348
Fasting blood glucose (mg/dL)	91	73-109
Albumin (g/dL)	3.4	4.1-5.1
Urea (mg/dL)	9.6	8.0-20.0
Serum creatinine (mg/dL)	1.01	0.46-0.79
Serum sodium (mEq/L)	121	138-145
Serum potassium (mEq/L)	3.2	3.6-4.8
NH3 (μg/dL)	76	12-70
Total bilirubin (mg/dL)	0.4	0.4-1.5
AST (U/L)	35	13-30
ALT (U/L)	14	7-23
Thyroid-stimulating hormone (μIU/mL)	0.44	0.61-4.23
Thyroxin (free T4) (ng/dL)	1.27	0.76-1.65

**Figure 1:**
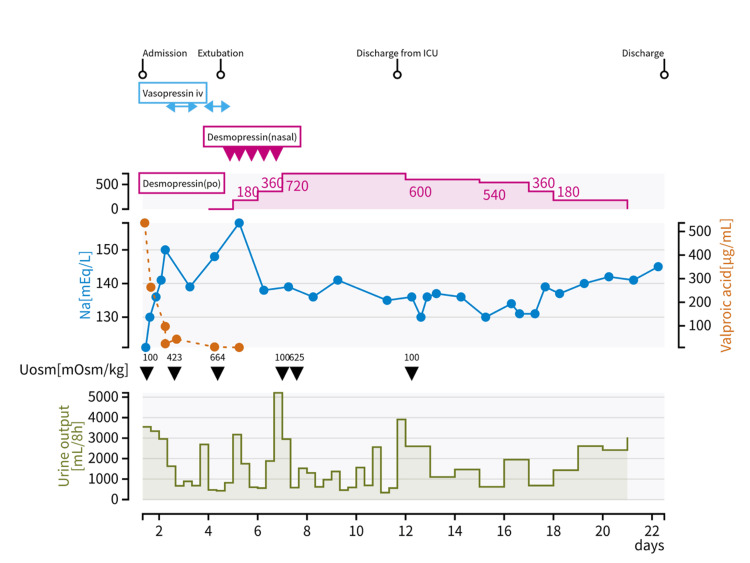
Course of urine output, sodium concentration, and urine-specific gravity during hospitalization The first row indicates events during hospitalization, with ICU standing for the intensive care unit. The second row shows administered medications, where “iv” represents intravenous injection, “nasal” indicates nasal drops, and “po” refers to oral medicine. The third row includes the left axis for blood sodium concentration and the right axis for blood valproic acid concentration. The fourth row displays urine osmolality. The fifth row represents urine volume every eight hours.

Owing to hypoxemia and impaired consciousness, the patient was intubated and admitted to the intensive care unit. Her serum valproic acid level was significantly elevated at 536.2 μg/mL (normal range, 50-100 μg/mL). She exhibited severely impaired consciousness and was in cardiogenic shock, with a heart rate of 97 bpm and blood pressure of 87/58 mmHg under norepinephrine infusion at 0.2 μg/kg/min. Therefore, continuous hemodiafiltration (CHDF) was initiated for two days [4]. Additionally, L-carnitine supplementation was administered at 3 g/day for seven days. The course of urine output, sodium concentration, and urine specific gravity during hospitalization are presented in Figure 1.

During hospitalization, the patient developed aspiration pneumonia, which was treated with ceftriaxone (2 g/day). On day 2 of hospitalization, she regained consciousness and was able to follow commands. Post admission, she excreted dilute urine with an osmolality of 100 mOsm/kg at a rate of 1000 mL/h, and her sodium level increased from 127 to 134 mEq/L within 12 hours. Owing to persistent polyuria and a further increase in sodium concentration, continuous intravenous vasopressin was initiated; however, urine osmolality remained elevated (Table 2).

**Table 2:** Laboratory test results before and after vasopressin administration

Laboratory test	Before vasopressin	After vasopressin
Serum sodium	121 mEq/L	146 mEq/L
Serum potassium	3.2 mEq/L	4.4 mEq/L
Urine-specific gravity	1.003	1.013
Urine osmolarity	102 mOsm/L	423 mOsm/L

**Figure 2:**
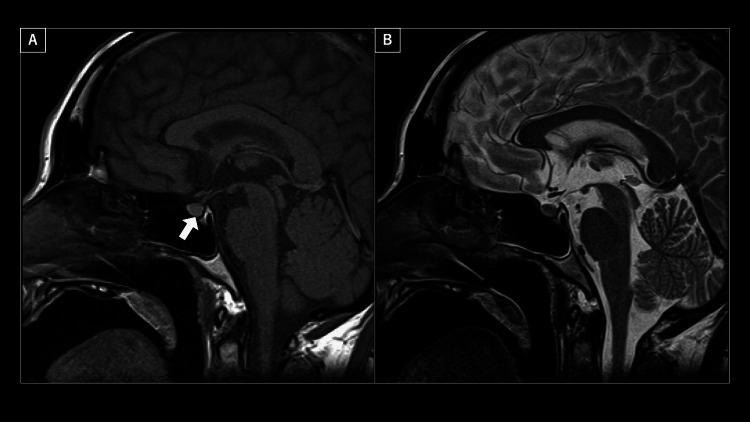
Sagittal cross-sectional MRI image of the head (A) T1-weighted image (arrow indicates loss of high signal in the posterior pituitary gland). (B) T2-weighted image.

Discontinuation of continuous vasopressin infusion led to increased urine output and elevated sodium levels, thereby requiring the initiation of intranasal desmopressin. The patient was extubated on day 4 of hospitalization, and oral desmopressin therapy was initiated. The increased urine osmolality post-vasopressin administration and the absence of a T1 hyperintense signal in the posterior pituitary gland in the head MRI (Figure 2) led to a diagnosis of AVP deficiency. There were no findings suggestive of cerebral edema on DWI or FLAIR head MRI images.

The initiation of oral desmopressin therapy reduced urine output and sodium levels, and the dosage was gradually reduced to prevent water intoxication due to hormone overreplacement. The patient did not experience polyuria recurrence, and treatment was successfully completed. The patient was discharged on day 24 of hospitalization. She was discharged home and has been attending her psychiatrist ever since.

## Discussion

In this report, we present the rare case of a patient who developed transient central diabetes insipidus during intensive care following impaired consciousness owing to an overdose of valproic acid. The patient had no history of diabetes insipidus and had not been treated with desmopressin.

While the possibility of AVP deficiency development before hospitalization was considered, it was unlikely since the patient had been unconscious for over 12 hours prior to admission and had been unable to drink fluids during this period. Additionally, as no treatment with desmopressin or similar medications was administered, AVP deficiency that developed before admission would have been accompanied by dehydration and hypernatremia due to the excretion of dilute urine, which was not observed. Furthermore, the finding of low sodium levels of 121 mEq/L suggested that polyuria onset occurred post-hospitalization.

Therefore, the cause of AVP deficiency in this case is believed to be related to an overdose of valproic acid. However, almost no reports of drug-induced AVP deficiency are available in drug information sheets or previously published studies [8]. AVP deficiency typically occurs due to trauma, pituitary tumors, infiltrative disorders, or infections [7]. Drug-induced diabetes insipidus occurs following the administration of medications such as lithium; however, it typically results from increased resistance to AVP and does not show responsiveness to AVP, as observed in this case.

The mechanism by which valproic acid causes central AVP deficiency is not well understood. Valproic acid is reportedly associated with the syndrome of inappropriate ADH secretion (SIADH) [9]. The findings, in this case, may be explained by either decreased sensitivity of osmoreceptors in the hypothalamus or inappropriate secretion of ADHs that eventually led to AVP deficiency in the posterior pituitary.

## Conclusions

In conclusion, we present a unique case of transient AVP deficiency that developed during hospitalization for the treatment of valproic acid overdose. Although the mechanism of injury to the posterior pituitary remained unclear, the patient gradually discontinued AVP supplementation, suggesting a reversible factor contributed to the condition.

Although SIADH due to valproate intoxication is a well-known complication, it is important to know that AVP deficiency can also occur, and prompt intervention can prevent harmful changes in Na concentrations and improve patient prognosis.
